# Ultracompact Nanophotonics: Light Emission and Manipulation with Metasurfaces

**DOI:** 10.1186/s11671-022-03680-w

**Published:** 2022-04-02

**Authors:** Yu-Heng Hong, Wen-Cheng Hsu, Wei-Cheng Tsai, Yao-Wei Huang, Shih-Chen Chen, Hao-Chung Kuo

**Affiliations:** 1 Semiconductor Research Center, Hon Hai Research Institute, Taipei, 11492 Taiwan; 2 Department of Photonics, National Yang Ming Chiao Tung University, Hsinchu, 30010 Taiwan

**Keywords:** Metasurfaces, Tunability, Reconfiguration, Structured light projection, Depth perception, On-chip, Monolithic integration, Light-emitting metasurfaces

## Abstract

Internet of Things (IoT) technology is prosperous for the betterment of human well-being. With the expeditious needs of miniature functional devices and systems for adaptive optics and light manipulation at will, relevant sensing techniques are thus in the urgent stage of development. Extensive developments in ultrathin artificial structures, namely metasurfaces, are paving the way for the next-generation devices. A bunch of tunable and reconfigurable metasurfaces with diversified catalogs of mechanisms have been developed recently, enabling dynamic light modulation on demand. On the other hand, monolithic integration of metasurfaces and light-emitting sources form ultracompact meta-devices as well as exhibiting desired functionalities. Photon-matter interaction provides revolution in more compact meta-devices, manipulating light directly at the source. This study presents an outlook on this merging paradigm for ultracompact nanophotonics with metasurfaces, also known as metaphotonics. Recent advances in the field hold great promise for the novel photonic devices with light emission and manipulation in simplicity.

## Introduction

Electromagnetic waves are present everywhere, especially in contemporary times with nowadays blossom of wireless, portable and wearable devices, and even of diverse consumer electronics. Accordingly, planar functional devices and systems for adaptive optics and spatial light modulators have been in the urgent stage of rapid development. Metasurfaces, ultrathin artificial structures at subwavelength scale, enable specific electromagnetic responses simultaneously *or* separately with amplitude, phase, and polarization [1–5]. Therefore, metasurfaces have achieved many astonishing results, including flat lens, holography, and optical vortex generation, and the like [6–9]. With proper design, metasurfaces can be efficiently adopted for manipulating these electromagnetic properties through light-matter interaction with the development of a generalized form of Snell’s law, and thus following the trend of miniaturization in relevant optical technologies, such as endoscopic imaging, computational imaging, optical information transmission and photonic imaging systems, and even the applications of augmented reality/virtual reality (AR/VR) for rapid emerging field of metaverse [10–22].

However, functionalities of static metasurface are decided right after fabrication. For modernistic and real-world applications, dynamic performances as well as miniature light manipulation are necessary. Miscellaneous strategies are employed to realize the dynamic performance for metasurfaces with tunability and reconfiguration [23–28]. Among these exploited strategies, with regard to the revolutionary metal-oxide-semiconductor field-effect transistors (MOSFETs) have profoundly affected the semiconductor industry and our daily lives nowadays, the electrical modulation with a metal-oxide-semiconductor (MOS) configuration can be a promising solution [29–31]. Therefore, several electrically tunable metasurfaces are implemented, and the corresponding electromagnetic responses can be dynamically controlled via applying different bias voltages [32–39].

Besides, the trend of miniaturization and the monolithic integration in relevant optical technologies become more and more prevalent, especially for the portable consumer electronics and the rapidly emerging demands on edge computing. Remote sensing techniques, namely the depth perception, become more and more crucial for machine vision and intelligence [40]. Thus, structured light projection plays an important role as the most commonly exploited approach for depth perception [41–43]. These structured light patterns are directly projected onto the target of interest. Thereby, the distortion of reflecting light patterns can be captured by an infrared camera and the corresponding depth information can be analyzed and extracted according to the geometric contours, and thus the relevant depth for target of interest. Compared to the conventional vision recognition, structured light projection for depth perception can operate in the dark environment, providing a much reliable approach in certain operating environment.

**Fig. 1:**
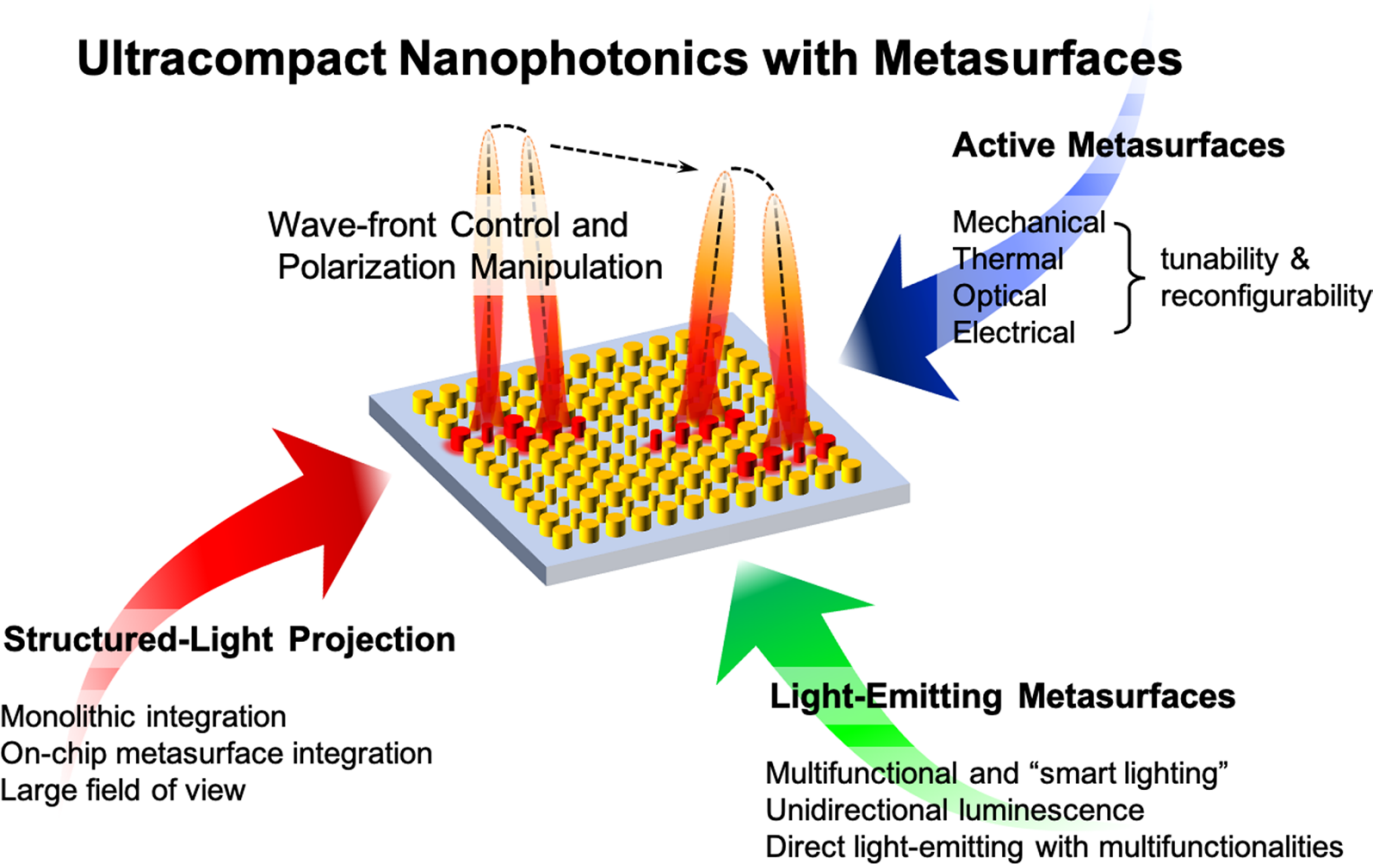
Schematic organization of relevant topics in this review

Moreover, integrating exotic optical and photonic components into the electronic systems not only can further boost the original capacities but also realize multitudinous functionalities, as the novel development of quantum information processing and communications with the trend [44]. Mercifully in this regard, metasurfaces are capable of mass production in wafer level with the complementary MOS fabrication compatibility, and thus an on-chip generation of structured light projection is then implemented with the modern monolithic integration, making these flat-profile artificial structures prospective candidates for an ultracompact meta-device. However, the separation of light-matter interaction, namely these fabricated artificial structures, and the light-emitting materials is still remained. Opportunely, several attempts and even for the field of nonlinear optics are proposed, demonstrating direct light-emitting metasurfaces with arbitrary functionalities [45–51]. Thus, with these efforts, the anticipation toward ultracompact nanophotonics with metasurfaces for structured-light projection in simplicity can be expected in the near future, as shown in Fig. 1.

## Basic Mechanisms for Reconfigurable and Tunable Metasurfaces

Herein, we overview the recent development of tunable and reconfigurable metasurfaces in different mechanisms as well as their advantages and disadvantages.

### Mechanical Tunability

**Fig. 2:**
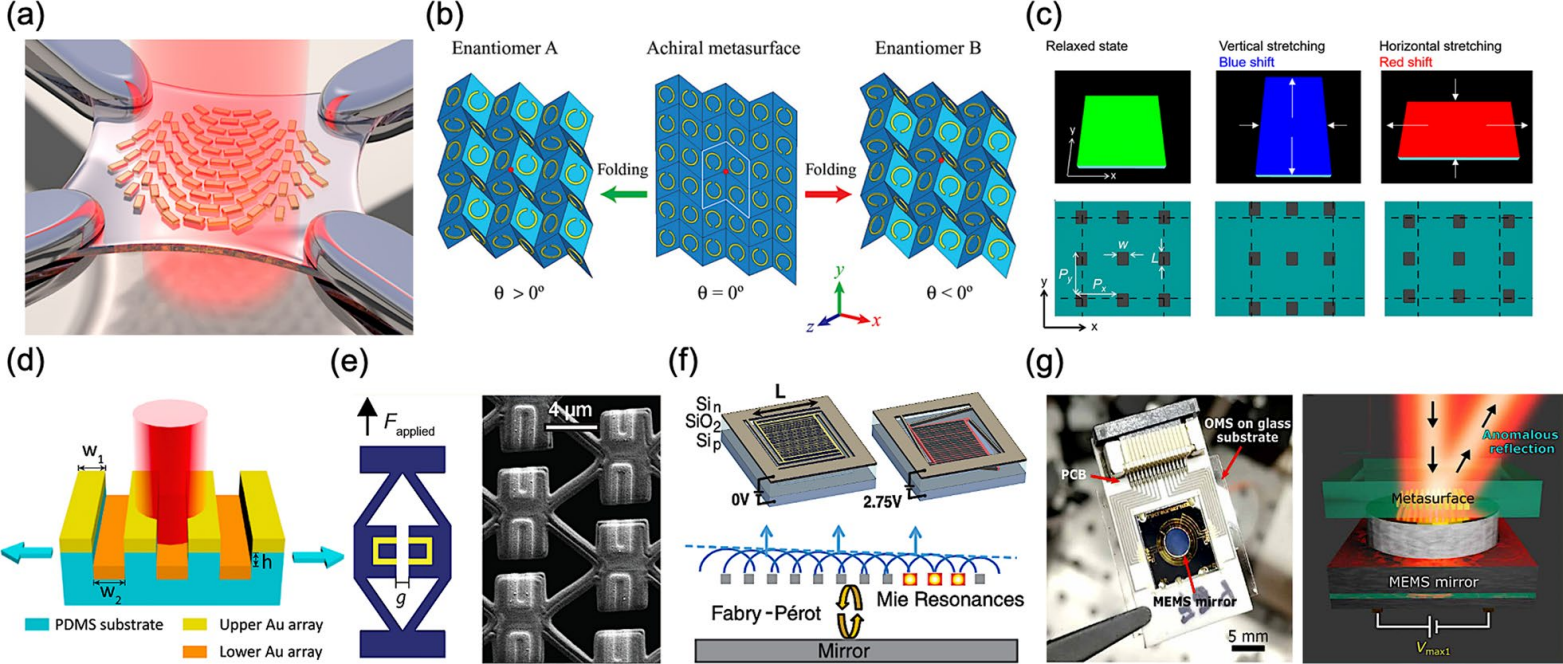
Mechanically reconfigurable and tunable metasurfaces **a** Tunable metasurface on a stretchable substrate [52], **b** Construction of Miura-ori chiral metamaterials [53], **c** Corresponding color changes under different stretching conditions [54], **d** Out-of-plane designed soft metasurface [55], **e** Mechanical metamaterials combined with optical meta-atoms (left), Corresponding SEM image (right) [56], **f** Temporal color control from suspended silicon metasurfaces (top), Resonance interaction between the Fabry-Pérot cavity and the nanowire Mie resonances (bottom) [57], and **g** MEMS-based optical metasurfaces assembly (left), Schematic of demonstrated anomalous reflection by bringing the MEMS mirror (right) [58]

For a conventional metasurface, its specific electromagnetic responses are determined by the shape, size, and geometric arrangements of each subwavelength element. Once the structural configuration is deformed by an external excitation, the corresponding electromagnetic responses can be changed, realizing the dynamic performance for a metasurface with tunability and reconfiguration. Hence, while a metasurface is fabricated on a flexible substrate for instance, the most intuitive way to regulate the eventual performance will be structural deformation via bending, stretching, folding, and rolling as a consequence of the changed constituent elements. In 2016, H. S. Ee et al*.* demonstrated a mechanically reconfigurable metasurface at the wavelength of 632.8 nm in visible range, as shown in Fig. 2a [52]. The designed metasurface is composed of a complex gold (Au) nanorod array fabricated on a stretchable polydimethylsiloxane (PDMS) substrate. Therefore, the wavefront can be continuously tuned via mechanically stretching, realizing in a continuous change of focal length from 150 to 250 μm, providing a potential way for reconfigurable flat optics with prospects.

In 2017, Z. Wang et al*.* reported the origami-based metamaterials whose electromagnetic responses can be dynamically controlled via switching the folding state of Miura-ori split-ring (C-shaped) resonators, as shown in Fig. 2b [53]. They experimentally observed different chiral responses by controlling the deformation direction and kinematics, opening a new avenue toward metadevices with simultaneously customized electromagnetic and mechanical properties. M. L. Chen et al*.* demonstrated a plasmonic metasurface for reflective color pixels, integrating a square array of aluminum nanostructures into an elastomeric PDMS substrate, as shown in Fig. 2c [54]. By means of mechanically stretching the substrate in either of its two dimensions, the period of nanostructural array can be modified; therefore, the corresponding scattering color can be tuned to the blue or the red of the at-rest structure, covering the entire visible spectrum.

In 2018, X. Liu et al*.* proposed a metasurface with an out-of-plane (non-coplanar) design on the PDMS flexible substrate, manifesting a reconfigurability of changing the resonance wavelength and energy of surface plasmon polaritons (SPPs) bloch wave, as shown in Fig. 2d [55]. In their proposed structure, the two-layered gold nanoribbon arrays can be mechanically changed via stretching its PDMS substrate, and thus the corresponging coupling plasmonic characteristics in between can be tuned and varied from the visible to near-infrared wavelength range, providing a promising revolution for current plasmonic devices. J. B. Reeves et al*.* proposed a tunable infrared metasurface on a soft microstructured polymer scaffold, using a micro-electro-mechanical-system-based (MEMS-based) stencil lithography technique, as shown in Fig. 2e [56]. Once applying force to the object holder, the deformation of polymer scaffold can be engineered, thus the relevant reflectivity in mid-infrared can be tuned, resulting a mechanical reconfigurable metasurface.

Even despite mechanical deformation is the most intuitive way to achieve the dynamic performance for a metasurface, modulating specific electromagnetic responses, the relevant mechanisms and corresponding mechanical control systems are quite complex and complicated for such designs. Moreover, these external excitations imposed on structural deformations are indirect and roundabout, hence it is difficult to accomplish a precise and real-time control for specific electromagnetic responses.

Herein, the main bottleneck in the way of mechanical deformation is the very limited modulation speed. Auspiciously, integrated relevant metasurfaces with MEMS can be a prospective method. In 2019, A. L. Holsteen et al*.* employed MEMS with silicon antenna arrays, realizing dynamic beam shaping of silicon metasurfaces [57]. The proposed metasurfaces can achieve a rapid response up to 100 kHz. Moreover, this platform technology exhibits a low-voltage operation of pixels for temporal color mixing and a dynamic beam shaping.

In 2020, C. Meng et al*.* combined a thin-film piezoelectric MEMS with a gap-surface plasmon-based metasurfaces for realizing complex dynamic two-dimensional (2D) wavefront manipulation [58]. The demonstrated platform exhibits a polarization-independent beam steering performance with fast responses down to 0.4 ms. Furthermore, the modulation efficiencies are around 50% with a broadband operating wavelength range up to 800 nm, offering flexible solutions in reconfigurable and adaptive optical networks and systems.

### Thermal Tunability

In addition to mechanical deformation of structural configuration for each subwavelength element, the change of material properties can be another feasible alternative [59–62]. Phase change materials (PCMs) possess inherently foreign material properties, which can be manipulated and switched with the change of operating temperature, optical excitation, carrier injection, or even mechanical force strain. Thus, PCMs can be exploited as a part of structure or an entire layer in the configuration, attaining the dynamic performance for a metasurface with tunability and reconfiguration. Among many PCMs, both vanadium dioxide (VO_2_) and germanium-antimony-tellurium (Ge:Sb:Te) (GST) alloy are widespread materials in temperature-responsive applications.

For VO_2_, relevant material state can change from a dielectric material to a metal, namely it can undergo from an insulating phase to a metallic phase, or vice versa. Owing to the low transition threshold in material state changing, VO_2_ is widely studied and adopted in thermally driven applications. The low transition threshold temperature of VO_2_ at 341 K (∼ 68 °C) makes it attract much attention in versatile applications [63]. As the common memory applications of PCMs, in 2009, T. Driscoll et al*.* proposed a hybrid system of memory metamaterials with split-ring resonators on a VO_2_ thin film [64]. While applying a voltage pulse on the electrodes, the hysteretic resistance behavior and a clear redshift of resonance can be observed within a complete temperature cycle; therefore, a novel form of memory capacitance is demonstrated.

**Fig. 3:**
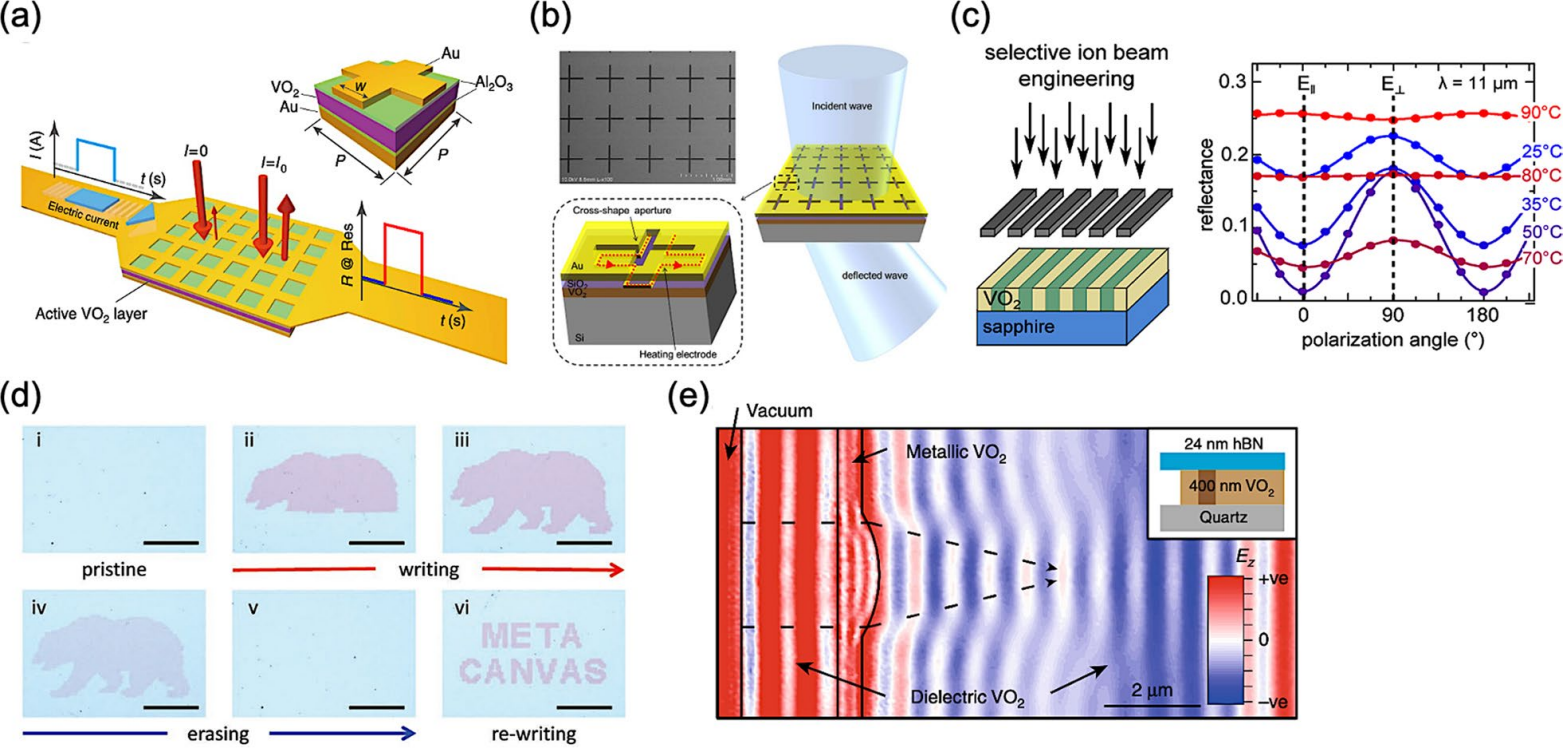
Thermally reconfigurable and tunable metasurfaces with VO_2_ **a** Meta-device consisting of an insulator-to-metal transition material [65], **b** Beam-steering metasurface with current-induced heating electrode [66], **c** Metasurface based on selective defect engineering of phase transition material [67], **d** Relevant writing and erasing process on a rewritable metacanvas [68], and **e** Simulation of a refractive polariton lens with phase-change material [69]

In 2016, L. Liu et al*.* proposed the hybrid metamaterials for electrically triggered multifunctional control, resulting in a meta-device with switchable reflectance, as shown in Fig. 3a [65]. Herein, the active layer of VO_2_ thin film is cladded within two alumina (Al_2_O_3_) dielectric layers, together with a bottom Au ground plane layer and a patterned-mesh top Au layer, forming a sandwich configuration. Accordingly, the patterned-mesh top Au layer not only supports optical resonances but also serves as electrodes connected to an external circuit for current injection, and thus generating Joule heating. Consequently, the corresponding reflection of incident light at normal incidence can be immediately tuned with different current injections. M. R. M. Hashemi et al*.* demonstrated a transmissive metasurface embedded with the phase-change material of VO_2_, possessing the capability of wavefront phase gradient engineering, as shown in Fig. 3b [66]. Such a reconfigurable metasurface can deflect the incident electromagnetic waves in an electronically controllable fashion. Moreover, the optimized metasurface can achieve a beam deflection angle up to 44° in both horizontal and vertical directions for operation at 100 gigahertz (GHz). J. Rensberg et al*.* demonstrated a spatially selective defect engineering based on nanometer-scale masks for metasurfaces with VO_2_ phase-transition material, as shown in Fig. 3c [67]. Utilizing the insulator-metal transition of VO_2_ along with this engineer technique, corresponding optical properties can be selectively controlled depending on its state, and thus a tunable metasurface can be fabricated for modulating the polarization state of light.

In 2018, K. Dong et al*.* presented an all-solid rewritable metacanvas, exploiting the hysteretic phase transition of VO_2_, as shown in Fig. 3d [68]. The entire writing process can be controlled below 90 °C using a low-power laser. Therefore, versatile physical (re)compilation of photonic operators can be compiled on the metacanvas for desired manipulation of light waveform akin to that of field-programmable gate array (FPGA). T. G. Folland et al*.* proposed a reconfigurable hyperbolic metasurface of isotopically enriched hexagonal boron nitride (*h*BN) directly contacted with single-crystal VO_2_, as shown in Fig. 3e [69]. Hence, the binary phase changes of VO_2_ in spatial, namely the metallic and dielectric states, can be utilized for modulating of polariton wave with reconfiguration.

Despite the material phase of VO_2_ can be reversibly changed via an external stimulus with a low transition threshold of temperature, the corresponding resonance shift and its spectral contrast are insignificant at near-infrared range, mostly in terahertz (THz) and infrared frequencies, thus limiting its practical applications. Nevertheless, the GST alloy, also a kind of PCM, can be a possible alternative [70, 71]. Typically, the phase transition of GST can change between amorphous and crystalline phases controlled by heating or cooling speed. At above melting temperture, a rapid cooling can prohibit from crystallization, and thus an amorphous state of GST can be obtained. Whereas one can conduct a sequence of heating process between melting temperature and glass transition temperature, thus a crystalline state of GST can be acquired. The most common applications of GST alloy in our everyday life are the fields of rewritable optical disk storage and nonvolatile electronic memories for its good thermal stability, fast-switching speed, and a reliable rewritability. Thus, by the integration with GST alloy, a dynamic performance for a metasurface with tunability and reconfiguration can be implemented.

**Fig. 4:**
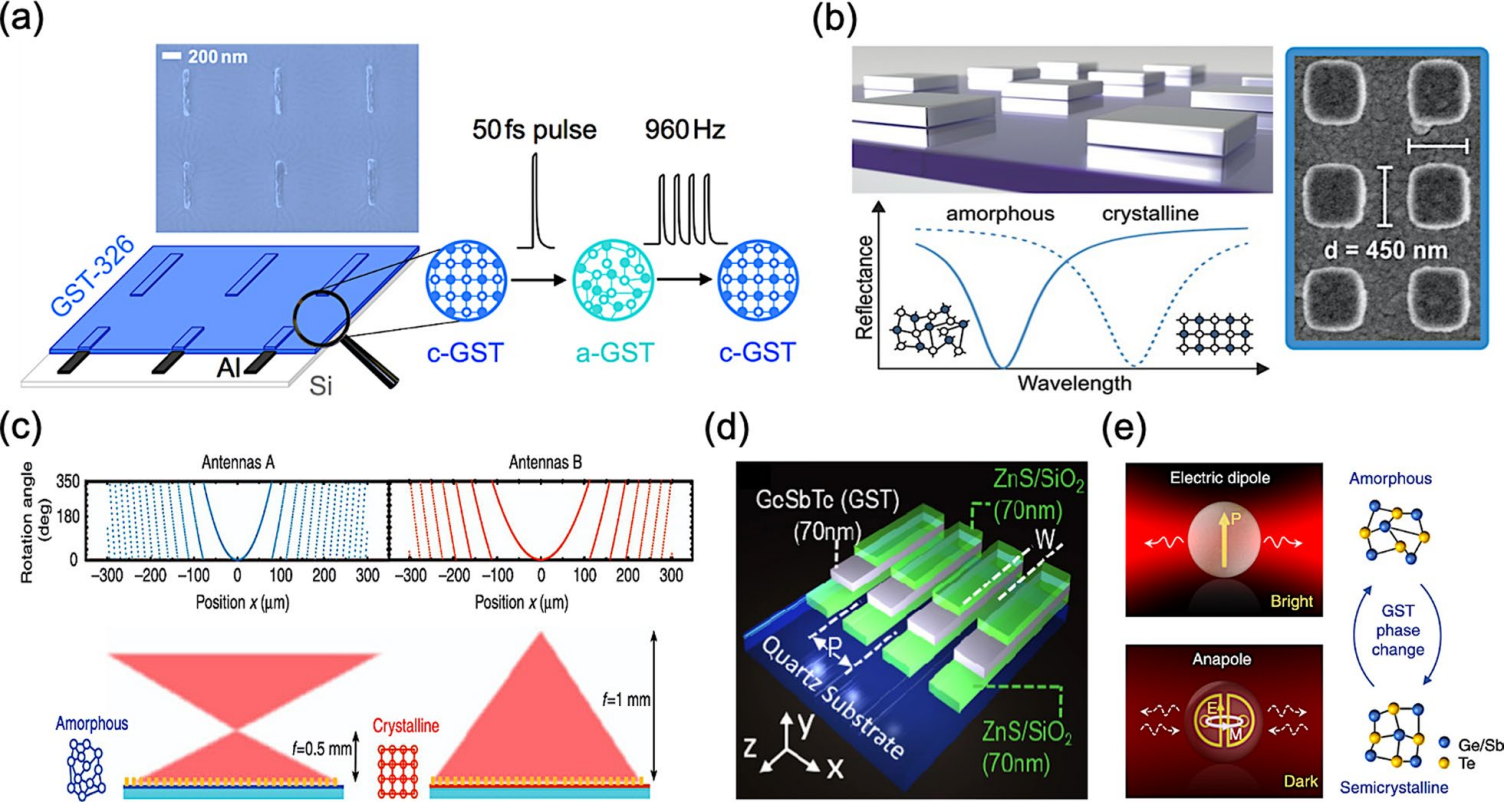
Thermally reconfigurable and tunable metasurfaces with GST **a** Reversible metasurface with nanoantennas controlled by femtosecond laser pulses [72], **b** Switchable perfect absorber meta-device with nanoantennas stacked above phase change material [73], **c** Cylindrical bifocal metalens for amorphous and crystalline GST [74], **d** Structures of a nanograting metasurface for dielectric-plasmonic transitions [75], and **e** Electric dipole resonance and an anapole excitation in a GST sphere introduced by an intermediate phase transition [76]

In 2014, A. K. U. Michel et al*.* presented a plasmonic antenna array with switchable optical properties, as shown in Fig. 4a [72]. In the configuration, the aluminum nanoantenna array is embed with an extremely thin GST film, and thus realizing a reversible optical switching of plasmonic resonances in the infrared range assisted by femtosecond laser pulses. In 2015, A. Tittl et al*.* proposed a switchable perfect absorber in mid-infrared range with multispectral thermal imaging capability, as shown in Fig. 4b [73]. The perfect absorber consists of an aluminum nanoantenna array stacked above an aluminum mirror with a spacer layer of GST. Thus, utilizing the material phase transition to provide strong reflectance contrast at resonance and a pronounced phase-change-induced spectral shifts of up to 25%, a band-selective performance with switchability can be achieved.

In 2017, X. Yin et al*.* presented a combination of resonant plasmonic metasurfaces with GST, demonstrating a opposite beam switching and bifocal lensing, as shown in Fig. 4c [74]. By interleaving two rows of antenna arrays with the dual-phase state of GST, such a metasurface can multiplex different functionalities due to different geometric phases, and thus resulting in a bifunctional device. In other words, for each phase state of GST, the resulted response of incident light is a consequence of one type of array. In 2018, B. Gholipour et al*.* implemented a phase shifter of a dielectric-plasmonic metasurface composed of chalcogenide with patterned GST nanostructures, as shown in Fig. 4d [75]. Tuning the material phase of GST via different external stimuli of a pulsed laser, such as its pulse number, repetition rate, and pulse energy, can induce a systematic conversion between dielectric and plasmonic states due to the change of GST dielectric constant. Thus, the consequent transmission and reflection characteristics, as changes in color, can be manifested, covering in the entire visible spectral range.

In 2019, J. Tian et al*.* demonstrated an active control of anapole states by structuring GST, as shown in Fig. 4e [76]. By changing the optical contrast of GST in amorphous and crystalline phases, the structured GST nanodisks can support a diverse set of multipolar Mie resonances with active tunability, realizing a broadband mode shifting between an electric dipole resonance and an anapole state. Moreover, in high order anapoles and multi-modal tuning, the structured GST can even serve as a multi-spectral optical switch with high extinction contrasts, which is larger than 6 dB.

Substantially, combined with diverse temperature-responsive materials, the dynamic performance for a metasurface can be implemented with a sufficient tuning range. The external stimuli for these tunable metasurfaces with thermally responsive materials can be typically a pulsed laser and even incorporated an integrated circuit [77]. However, in particular for VO_2_, the tuning spectral domains are typically outside of the visible range, mostly at THz and infrared frequencies, and thus limiting its practical applications. Besides, the thermal-driven modulation speed is still not enough for a precise and real-time control due to the required time for heating and cooling process.

### Optical Tunability

Due to photocarrier dynamics, specific materials can sufficiently change their optical properties in response to light by adjusting pumping light sources. Considering the required time of the dynamic performance for a metasurface with tunability and reconfiguration, optical excitation induced by the pumping light source is an effective and prompt modulation method on demand, exhibiting a rapid response within femtoseconds (fs) or picoseconds (ps) in typical. Herein, we mainly focus on semiconductor materials in controlling THz waves.

**Fig. 5:**
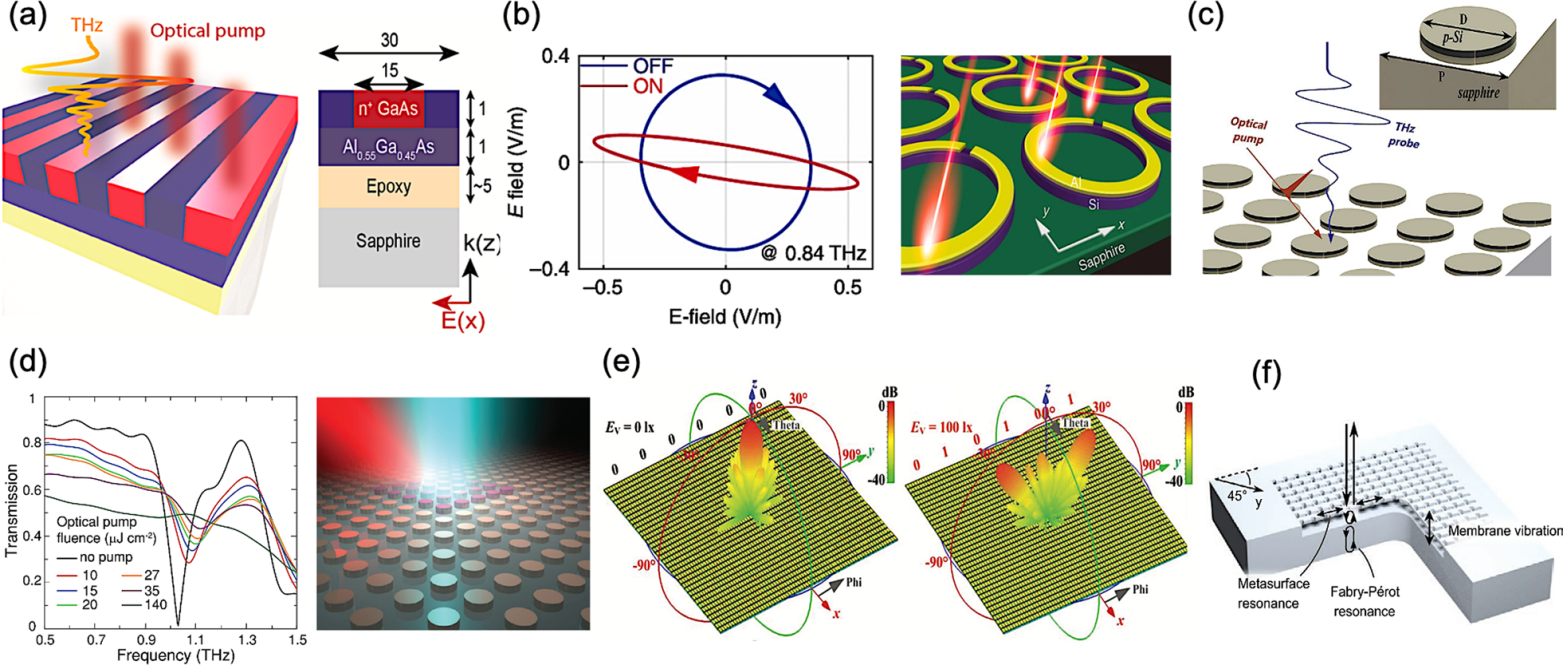
Optically reconfigurable and tunable metasurfaces **a** Transient GaAs metasurface induced by a structured femtosecond optical pump beam [78], **b** Calculated two output polarization ellipses in the “ON” and “OFF” states (left), Active control of anisotropy in *h*-SRR metasurface (right) [79], **c** Plasmonic metasurface controlled by an optical-pump terahertz-probe spectroscopy [80], **d** Measured transmission coefficient for different values of the optical fluence (left), All-dielectric metasurface pumped by an ultrafast laser beam (right) [81], **e** Simulated reflection beams of the light-controlled digital coding metasurface at 3.75 GHz with different coding sequences [82], and **f** Optomechanics of a chiral metasurface consisting of a semiconductor membrane patterned with L-shaped holes [84]

In 2017, Y. Yang et al*.* presented an ultrafast formation of THz metasurface using the III-V compound semiconductor materials of gallium arsenide/aluminium gallium arsenide (GaAs/AlGaAs) heterostructure, as shown in Fig. 5a [78]. Thus, the all-optical creation of spatially modulated carrier density profiles can be generated in in a deep-subwavelength GaAs film. The switch-on of the transient plasmon mode is revealed on a time scale of 500 fs. By adjusting different pumping fluences, the electric dipole resonance of the transient GaAs metasurface can be observed from 0.5 to 1.7 THz.

In 2018, L. Cong et al*.* proposed an all-optical active THz metasurface for ultrafast polarization switching and dynamic beam splitting, as shown in Fig. 5b [79]. In the configuration, a layer of silicon rings is between the C-shaped arrays, commonly used in the conventional metasurface, and a sapphire substrate. By adjusting the intensity of pumping light source, an ultrafasr modulation of polarization states and the beam splitting ratio can be experimentally acquired on a time scale of 667 ps. H. Cai et al*.* also demonstrated an all-optical active THz plasmonic metasurface for an ultrafast modulation of absolute transmission up to 38%, as shown in Fig. 5c [80]. The active metasurface is composed of an ion-implanted and annealed silicon disk arrays. By utilizing the THz pump-probe spectroscopy, corresponding electric dipole resonance in the silicon-based configuration can exhibit a switch-on time within 20 ps and a recovery time of 300 ps.

In 2018, K. Fan et al*.* proposed a phototunable dielectric Huygens’ metasurface, manipulating the resonant eigenmodes through an optical excitation, and thus overcoming the static geometrical tuning characteristics of a conventional dielectric metasurface, as shown in Fig. 5d [81]. Thence, with this silicon-based dielectric configuration, an intensity transmission modulation depth of 99.93% at 1.03 THz with an associated phase change of π/2 rad can be achieved under an optical pumping fluence of only 10 µJ cm^−2^. X. G. Zhang et al*.* proposed a remote-mode metasurface tuned by illuminating light, exhibiting a digital coding tunability in a control-circuitry-free way, as shown in Fig. 5e [82]. By modulating the intensity of integrated photodiodes, such light-addressable elements in the configuration can dynamically implement reconfigurable radiation beams at microwave frequencies, namely at the end of THz band, demonstrating an unprecedented digital metasurface of noncontact remote fashion. However, the adoption of additional photodiodes to achieve a reconfigurable function is bulky, forfeiting the benefit of a flat-profile metasurface, and thus increasing the complexity to some extent.

In the recent years, a novel fashion of metasurfaces, exploiting the nonlinear characteristics of optomechanics, has attracted much attention as well [83, 84]. Besides, in 2020, S. Zanotto et al*.* presented a chiral dielectric metasurface, as the first example of “Polarization Optomechanics”, giving an access to new forms of polarization nonlinearities and control, as shown in Fig. 5f [84]. With the excitation of an incident electromagnetic wave, the mutual coupling effect between nanoscale mechanical motion and optical chirality of structure can accordingly affect and modulate the self-polarization state.

The use of optically driven elements for a tunable metasurface can achieve a rapid response for an ultrafast modulation by simply adjusting the pumping light sources. However, the main disadvantages include a relative narrow tuning range and the unfeasibility of precisely controlling each element, yet usually for the entire metasurface.

### Electrical Tunability

To realize an electrically driven metasurface with tunability and reconfiguration, two main methods have been widely explored. Just as the most intuitive way of the aforementioned structural deformation for a metasurface fabricated on a flexible substrate, loading electrically controlled components with the constituent elements of a metasurface can be a straightforward strategy as well. Hence, a voltage-controlled metasurface can be acquired by adjusting different bias voltages for the loaded elements, exhibiting simple tuning abilities. Besides, integrating an electrically tuning material with a proper designed metasurface can be another direct method to achieve an electrical tunability for the metasurface as well. Via applying static electric fields, namely changing the corresponding carrier density, the material properties of the integrated material can be tuned, and thus the corresponding electromagnetic responses of such a metasurface can be dynamically controlled.

**Fig. 6:**
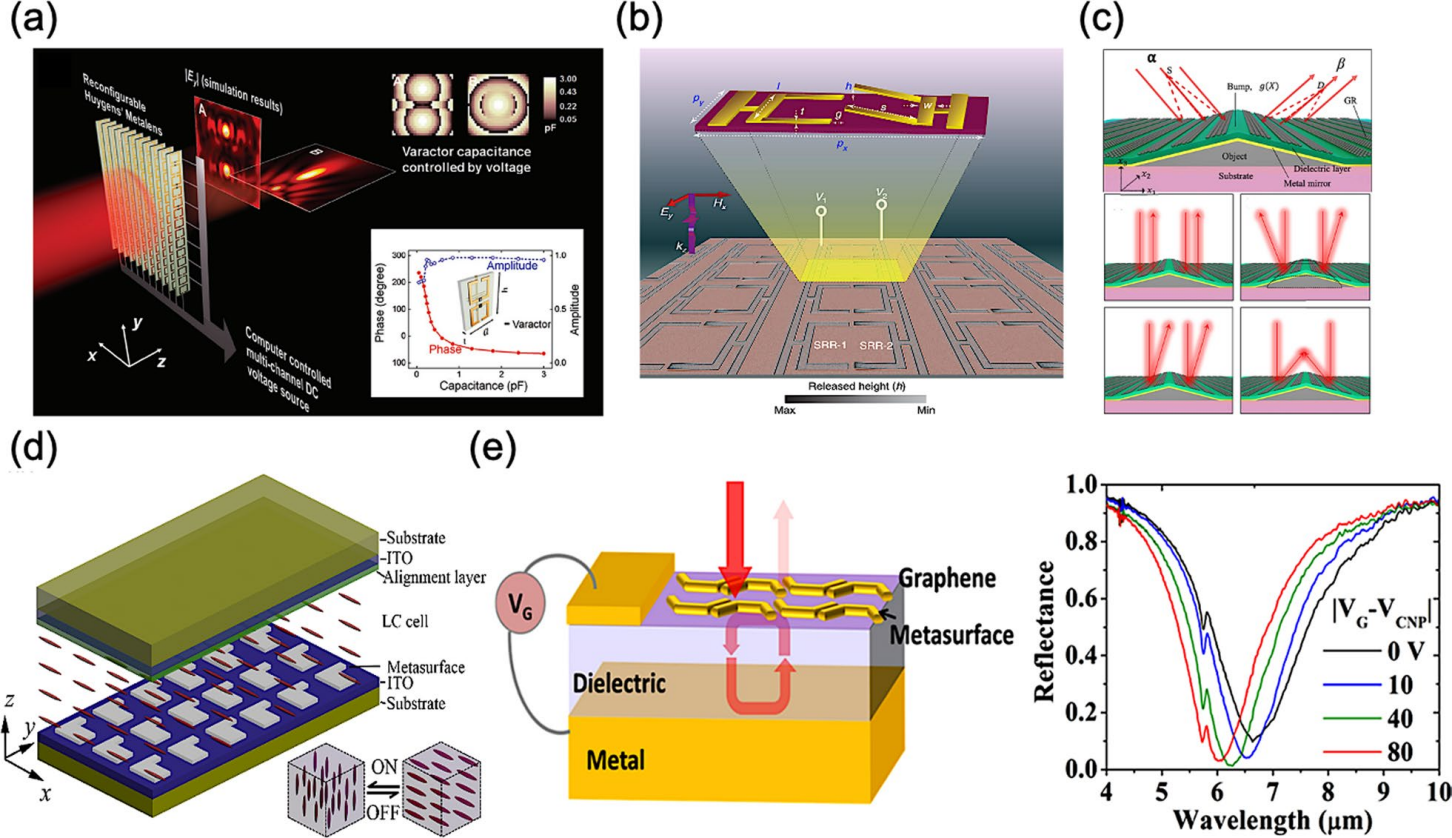
Electrically reconfigurable and tunable metasurfaces **a** Active Huygens’ metalens controlled by multichannel DC voltage sources [85], **b** MEMS Fano metasurface with multiple-input–output states for logic operations [86], **c** Metasurface with graphene ribbon array on a nonplanar surface [87], **d** Liquid–crystal-loaded chiral metasurfaces for reconfigurable multiband spin-selective light absorption [88], and **e** Ultrathin optical modulator based on a tunable metasurface absorber operating in mid-infrared range [89]

In 2014, Y. Yao et al*.* proposed an electrically tunable metasurface perfect absorber, achieving a modulation depth of up to 100% over a broad wavelength range (from 5 to 7 μm), as shown in Fig. 6a [85]. The designed metasurface arrays on graphene are incorporated with into a subwavelength-thick optical cavity, and thus the absorber in and out of the critical coupling condition can be manipulated with different gate voltages applied to graphene layer.

In 2017, A. Komar et al*.* demonstrated a liquid–crystal-loaded chiral metasurface, resulting in a spin-selective absorption of a single-layered metasurface with controlled resonance frequency, as shown in Fig. 6b [86]. By integrating liquid crystals on to metasurface arrays, relevant absorption on demand for the incident light with different polarizations can be selected via regulating bias voltages applied to the liquid crystals. Thus, a variance of absorption efficiency, up to 40%, can be achieved between the left- and right-hand circular polarization state of incident light. K. Chen et al*.* reported a Huygens’ metasurface at the microwave frequencies, loading with controllable active elements, as shown in Fig. 6c [87]. For such a reconfigurable metasurface array, the consequent electromagnetic responses can be tuned by manipulating different bias voltages with fast response time and high efficiency, exhibiting a real-time electromagnetic wavefront control. Thus, for the first time, multiple and complex focal spots can be simultaneously controlled.

In 2018, S. R. Biswas et al*.* presented a tunable metasurface composed of graphene ribbon arrays, as shown in Fig. 6d [88]. In the simulation, they proved such a metasurface exhibits an on-demand switchability between different regimes of operation by adjusting different bias voltages for the graphene ribbon arrays. Furthermore, the designed metasurface can realize versatile capabilities in optics, such as anomalous beam steering and focusing, cloaking, and illusion optics. M. Manjappa et al*.* experimentally demonstrated a reconfigurable MEMS-loaded Fano metasurface at THz frequencies, performing multiple-input–output states for simple logic operations, as shown in Fig. 6e [89]. Thereupon, in the different regions of electromagnetic field, distinct behaviours of Fano resonance can be observed. The corresponding Fano resonance enables XOR and XNOR operations in the far field, while a NAND operation can be observed in the near field.

The aforementioned mechanisms can achieve the dynamic performance for a metasurface with tunability and reconfiguration; however, an ultimate metasurface with integration and scalability is still remained, considering the manufacture on a mass-production basis and the bulky form factor of status quo for an integrated circuit. Nevertheless, with regard to the development of contemporary semiconductor technology, bulky form factor of junction transistors, such as bipolar junction transistors (BJTs), is solved by the invention of field-effect transistors (FETs) [29]. A FET can dynamically control the current flow in semiconductor via applying an electric field to the gate and meanwhile presents the ease of integration and scalability. Afterwards, a breakthrough in FET research comes in the late 1950s, introducing a thin silicon oxide layer on silicon surface to neutralize surface states, namely the surface passivation [30]. Thus, this practical technique makes the mass production of silicon-based integrated circuits possible. Thereafter, the MOSFETs profoundly affect the semiconductor industry and our daily lives, becoming the most ubiquitous type of transistor in consumer electronics nowadays. Recently, in 2019, the United States Patent and Trademark Office even called the MOSFET a "groundbreaking invention that transformed life and culture around the world" [31].

Thence, the prominent MOS configuration can be exploited as a reliable method to provide the static electric field for an electrically tuning material, namely an active semiconductor layer. Typically, a hybridized metasurface for tunability and reconfiguration exploiting a MOS configuration may possess a metal-insulator-metal (MIM) structure, namely a metal-transparent conducting oxide (TCO)-semiconductor heterostructure. In this architecture, TCO material, namely a heavily doped metal oxide as the semiconductor material, can thus serve as an active layer. As the depiction in Drude model, bulk plasma frequency depends on the density of free carriers, and thus the manipulation of doping concentration, namely the carrier density, can be adopted for acquiring specific electromagnetic responses at desired frequencies [90]. Owing to the heavily doping concentration of TCO materials, abundant plasmonic responses can be observed as well [91]. Therefore, Drude model can be employed for depicting the relevant properties of TCO materials, and thus describing the relevant plasma frequency and the corresponding permittivity. Moreover, in the visible and near-infrared region, TCO materials can be great alternative plasmonic materials due to a much lower loss than that of conventional metalic materials, such as gold and silver [91–94].

For the typical MOS configuration of a hybridized metasurface, while sufficient bias voltage is applied to the metallic electrodes, numerous carriers larger than the intrinsic doping concentration will accumulate at the interface between dielectric and TCO material. Thereby, this field-effect-induced accumulation can be exploited for an electrical modulation using the large variation of in situ electric field, and thus for the refractive index [95]. Furthermore, an epsilon-near-zero (ENZ) effect occurs when the real part of permittivity in TCO material changes from a positive value into a negative value; therefore, strong electromagnetic confinement can be observed in the accumulation layer, resulting in an enhanced optical performance around the interface [96, 97]. To sum up, electrically manipulating the carrier density of TCO materials can modulate the corresponding permittivity on demand, and thus resulting in a change of refractive index for specific electromagnetic responses.

**Fig. 7:**
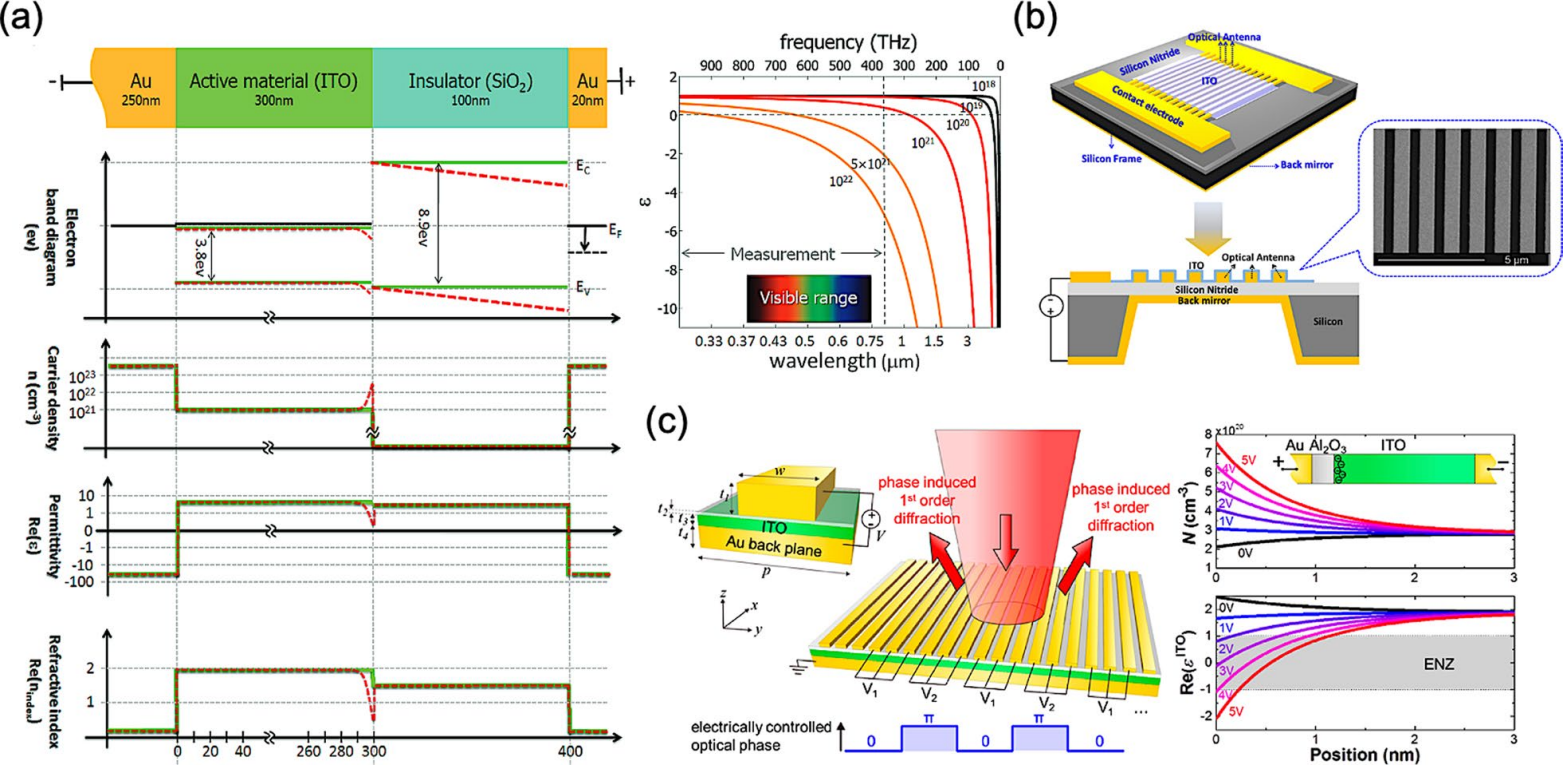
Electrically reconfigurable and tunable metasurfaces **a** Unity-order index changing in MOS structure with ITO material in visible range [98], **b** Voltage-tuning metasurface absorber with plasmonic nanostrip antennas [99], and **c** Gate-tunable metasurface with dynamic phase grating [32]

Among diverse TCO materials, such as indium-tin oxide (ITO), doped zinc oxide (Al:ZnO and Ga:ZnO), and doped cadmium oxide (CdO:In), etc., ITO plays important roles, especially for the applications in the near-to-mid-infrared wavelength range and even in visible wavelength range. It is worth mentioning that, in 2016, M. Z. Alam et al*.* observed a huge change in refractive index around 0.72 of ITO material operated in the ENZ region, as shown in Fig. 7a [98]. Early in 2013, F. Yi et al*.* demonstrated a perfect plasmonic absorber in mid-infrared range with the voltage tunability, as shown in Fig. 7b [99]. In the proposed configuration, gold nanostrip antennas are separated from a back reflector by a thin dielectric layer. And the ITO material, as the active layer, covering on the nanostrip antennas is employed to achieve a spectral tunability via applying different bias voltages.

Typically, MIM structures have been widely adopted for the conventional reflective plasmonic metasurfaces [100, 101]. Therefore, utilizing the field-effect-induced accumulation, MIM structures combined with ITO material can achieve an electrical modulation. Miscellaneous types of gate-tuable metasurfaces with dynamic performance are thereby proposed [32–34]. In 2016, Y. W. Huang et al*.* demonstrated a representative top-gate tunable metasurface with full use of aforementioned mechanisms, as shown in Fig. 7c [32]. The relevant MOS configuration formed on a quartz substrate consists of a top gold stripe nanoantenna array, a thin Al_2_O_3_ film, a thin ITO layer as the accumulation layer, and a bottom gold back plane. Once the bias voltage is applied between the top gold stripe and the bottom gold plane, carrier accumulation occurs at the interface of Al_2_O_3_ and ITO, modulating the phase and amplitude of reflected light at mid-infrared range on demand. With the similar concepts, A. Forouzmand et al*.* designed a an electrically tunable reflectarray metasurface for 2D beam steering [35]. Top square-shaped patch nanoantennas are placed on a stack of insulator-ITO-metallic ground plane, therefore forming a MIM structure. Thus, the resonant characteristics can be electrically manipulated via varying bias voltages, obtaining over 250° of phase agility at around 218 THz.

**Fig. 8:**
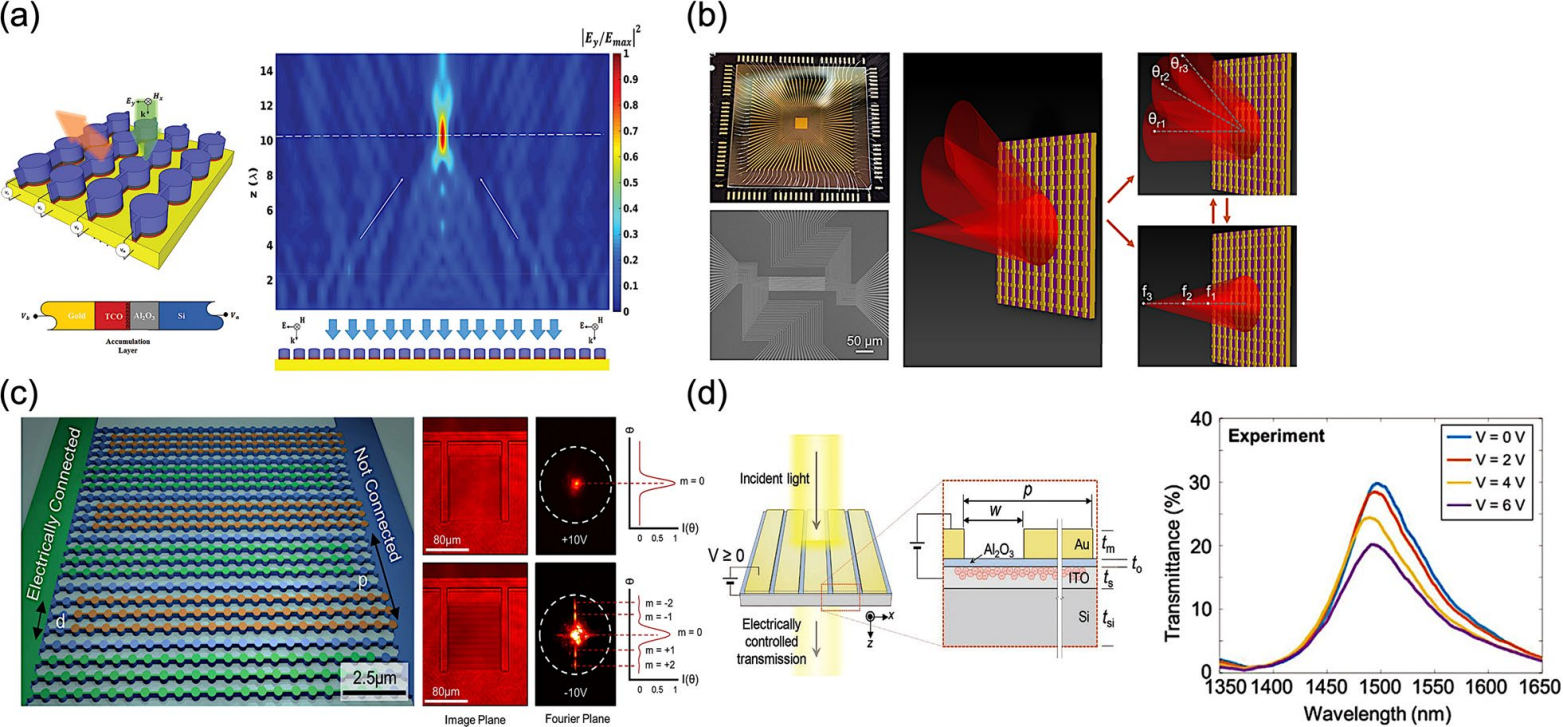
Electrically reconfigurable and tunable metasurfaces **a** Tunable ITO-assisted metasurface as a reflective beam steering platform [36], **b** Multifunctional metasurface with 96 independently addressable metasurface elements [37], **c** All-dielectric Huygens metasurface for dynamic transmission control [38], and **d** Gate-tunable metasurfaces for dynamic transmission control with hybrid plasmonic waveguide mode [39]

Soon after in 2018, A. Forouzmand et al*.* once more demonstrated a tunable multi-gate ITO-assisted all-dielectric metasurface in near-infrared wavelength range, as shown in Fig. 8a [36]. Hence, an optically active reflective metasurface can be achieved owing to the resonances in a silicon-made nanoantenna arrays and thus the use of carrier accumulation at the interface between dielectric and TCO material, namely ITO herein, under the influence of external multigate biasing. Consequently, taking advantage of different voltage biasing, this tunable and reconfigurable reflectarray metasurface can achieve diverse functionalities, such as a linear or circular polarizer, a dynamical beam steering device, and a a tunable metalens with controllable focusing patterns.

Regarding the aforementioned functionalities, the complexity of voltage biasing networks will play important roles in the tunability and reconfiguration of a hybridized metasurface. Thus, toward a more sophisticated modulation, in 2020, G. K. Shirmanesh et al*.* made a step forward, as shown in Fig. 8b [37]. A multifunctional metasurface with independently array-level constituent elements up to 96 is demonstrated, and thus accomplishing arbitrary optical functions, such as dynamic beam steering and reconfigurable light focusing.

In aforementioned MIM architectures, a reflectarray metasurface often include metallic resonators; therefore, unwanted absorption loss will occur, limiting the overall device efficiency with a severe power consumption. In 2018, A. Howes et al*.* proposed an all-dielectric Huygens metasurface with an on-state transmittance of 70% and a modulation depth of 31%, utilizing the ENZ mode of ITO thin films, as shown in Fig. 8c [38]. Based on this mechanism, a tunable diffraction grating is thereby demonstrated as well, providing a new avenue for beam steering applications with high-speed modulation and a low-power power consumption.

In 2020, Y. Lee et al*.* presented a gate‐tunable metasurface for the transmission control of incident light at near-infrared range using hybrid plasmonic waveguide modes to overcome the low modulation speed due to the high resistance of dielectric materials, as shown in Fig. 8d [39]. The adoption of hybrid plasmonic waveguide mode using ITO material thus enables a high switching speed with a large modulation depth, revealing a modulation speed of around 826 kHz experimentally.

## Metasurfaces for Structured Light Projection in Depth Perception

**Fig. 9:**
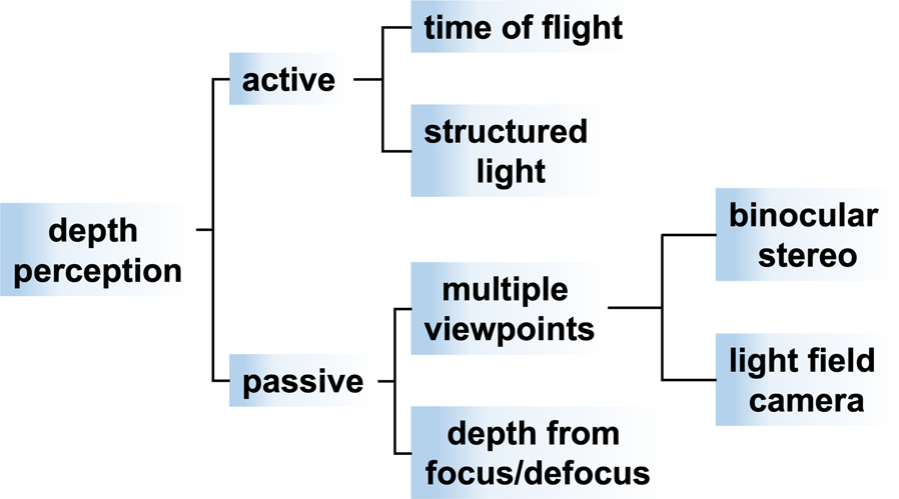
Categorization of depth perception

**Fig. 10:**
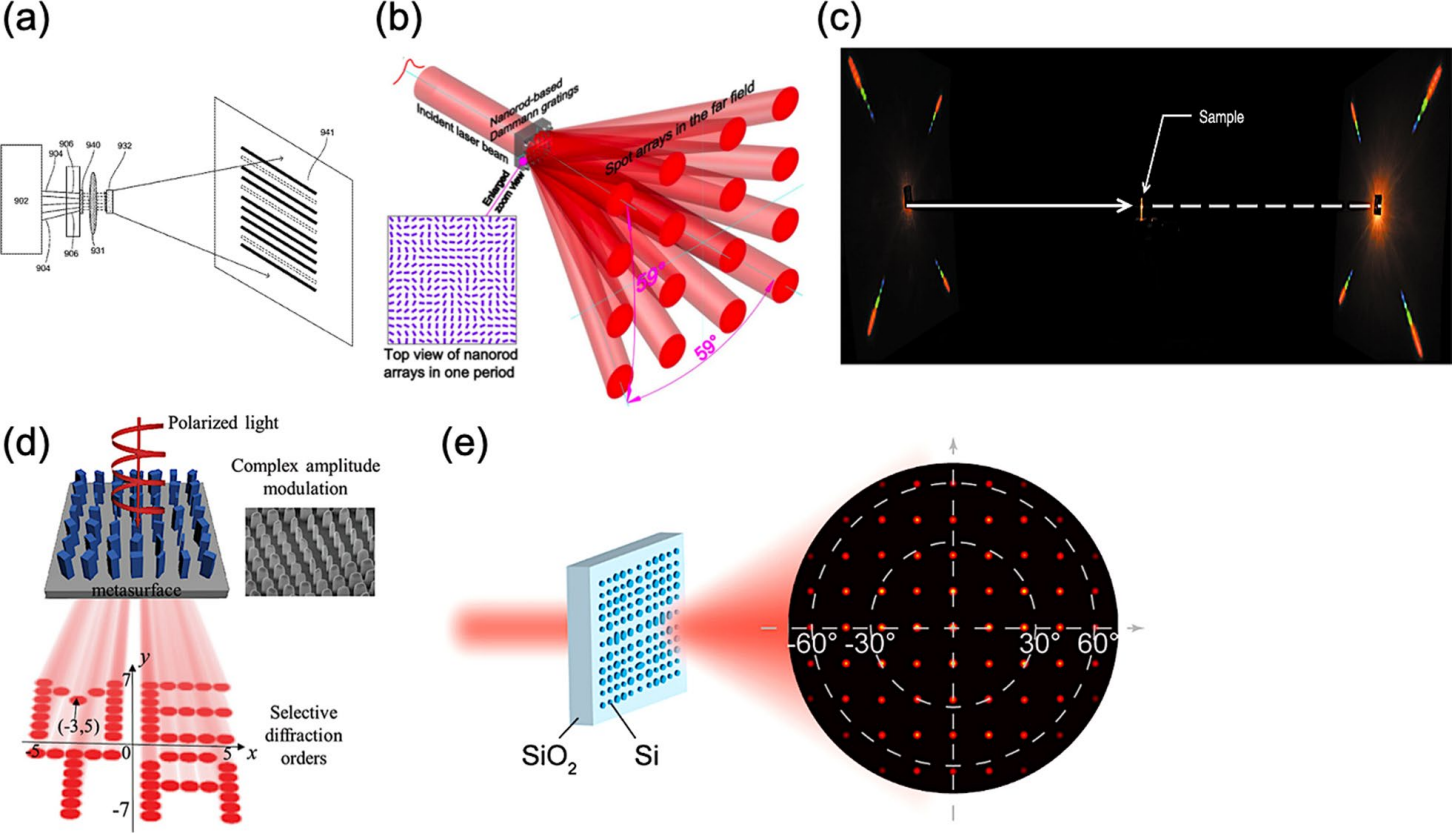
Structured light projection with metasurfaces **a** A typical structured light projector consisting of a light source, a collimator lens, and a diffractive optical element (DOE) [102, 103], **b** All-silicon nanorod-based Dammann gratings for splitting incident beam into spot arrays [122], **c** Full-space cloud of random points with a scrambling metasurface [123], **d** Complex amplitude modulation of the selective diffraction with a dielectric metasurface [124], and **e** Metasurface for structured light projection with 120° field of view [125]

Miscellaneous methods for depth perception have been proposed, herein, we mainly focus on the method of structured light projection in depth perception, as shown in Fig. 9. Typically, a depth perception system with structured light patterns comprises of a light-emitting device, a collimator lens, and a diffractive optical element (DOE), as shown in Fig. 10a [102, 103]. The light-emitting device can be a vertical-cavity surface-emitting laser (VCSEL) array with an adjoining collimator lens to obtain a desired beam diameter. Then, the DOE can further project and expand the collimated laser beam to obtain the designed structured light patterns with a large field of view (FOV). Therefore, while these structured light patterns are projected onto the target of interest, the distortion of reflecting light patterns can be captured by an infrared camera. Thus, according to the geometric contours and the relevant depth for target of interest, the corresponding depth information can be analyzed and extracted. Among miscellaneous gratings for a proper DOE, a Dammann grating, namely the binary-phase grating with a tailored transmission function, is widely adopted in the conventional depth perception system [104, 105]. Thence, exploiting the scalar diffraction theory with iterative algorithms for further optimization, the corresponding transmission function of the DOE can be designed, generating the desired light patterns in the far-field [106–108].

### From Diffractive Optical Elements to Metasurfaces

As yet, the relevant FOV of conventional DOEs is quite limited, approximately less than 60° × 60° in typical, due to the inaccuracy of the scalar diffraction theory with a small periodicity approaching to the wavelength scale of incident light. Specifically, to obtain a larger FOV, a reduced periodicity of the DOE is necessary to diminish the stray lights from undesired diffraction orders. However, while the periodicity of the DOE is comparable with the wavelength of incident light, the scalar diffraction theory will be inaccurate. Moreover, the phase modulation of a conventional DOE fundamentally relies on the relative difference of optical path length (OPL) for multiple phase levels, namely distinct etching depth, are required, increasing the complexity of fabrication.

Fortunately, the aforementioned limitation can be well solved with the rise of metasurfaces with flat-profile constituent structures. By adjusting the subwavelength elements of a metasurface, the corresponding electromagnetic responses, namely the amplitude, phase, and polarization, can be modulated [2, 10, 109–111]. In particular, compared to a conventional DOE, the phase modulation of a metasurface is achieved via resonance or waveguiding effects, thus providing great flexibility within a subwavelength spatial resolution in a flat-profile artificial structure [2, 5, 112–116]. Therefore, to acquire a large FOV, namely a large numerical aperture, metalenses are therefore adopted in depth perception, replacing the diffractive lenses [117–121].

Accordingly, for the selective diffraction, in 2015, Z. Li et al*.* proposed an all-silicon nanorod-based Dammann gratings to overcome the contradiction between the complexity of fabrication and the eventual performance of such gratings by introducing the concept of geometric phase, namely Pancharatnam-Berry phase, for phase modulation in depth, as shown in Fig. 10b [122]. Consequently, a uniform 4 × 4 spot arrays with an extending angle of 59° × 59° can be obtained in the far field. Moreover, the single-step fabrication procedure of identical etching depth can provide a more accurate phase modulation with a strong polarization conversion efficiency.

Soon in 2018, Z. Li et al*.* demonstrated a scrambling metasurface, generating a full-space cloud of random points compared to a typical Lambertian operation only in half space, as shown in Fig. 10c [123]. Hence, an astonishing number of random spots over 4044 at nearly 90° is experimentally observed in the entire space. X. Song et al*.* presented a dielectric metasurface, exhibiting the selective diffraction with a complex amplitude modulation, and thus can split the incident light into several beams with desired profile, as shown in Fig. 10d [124]. By spatially tailoring the geometric parameters of the subwavelength elements for such a metasurface, the corresponding phase as well as the amplitude can be simultaneously controlled.

However, the aforementioned metasurfaces may require a circularly polarized incident beam yet, or rely on scalar diffraction theory with iterative Fourier transform algorithm, as a binary-phase grating, to obtain a consequent transmission function for desired light patterns in the far-field. Besides, a significant portion of zeroth-order transmission can be experimentally measured, and thus severly hindering the practical applications. In 2020, Y. Ni et al*.* gave a thorough solution to overcome the limitations of conventional binary-phase Dammann gratings, employing vectorial electromagnetic simulation and interior-point method for the optimization, as shown in Fig. 10e [125]. Thus, a silicon-based metasurface is experimentally demonstrated with a fourfold rotational symmetry to eliminate the polarization dependence. For the telecommunication wavelength of 1550 nm, the designed metasurface can project a 2D spot array in the far-field with an exceedingly large FOV over 120° × 120°, maintaining a relatively high efficiency with the decent spot uniformity as well. Moreover, the metasurface consists of binary silicon patterns on a fused silica substrate, allowing mature nanofabrication techniques on a mass-production basis.

### On-Chip Generation of Structured Light Projection

The aforementioned emerging ultrathin flat-profile artificial structures, namely metasurfaces, can provide great flexibility and specific electromagnetic responses within a subwavelength spatial resolution. However, most of these demonstrated metasurfaces are separated from the electromagnetic sources, namely the light emitters, and require a proper irradiation for the designed optical functionalities.

**Fig. 11:**
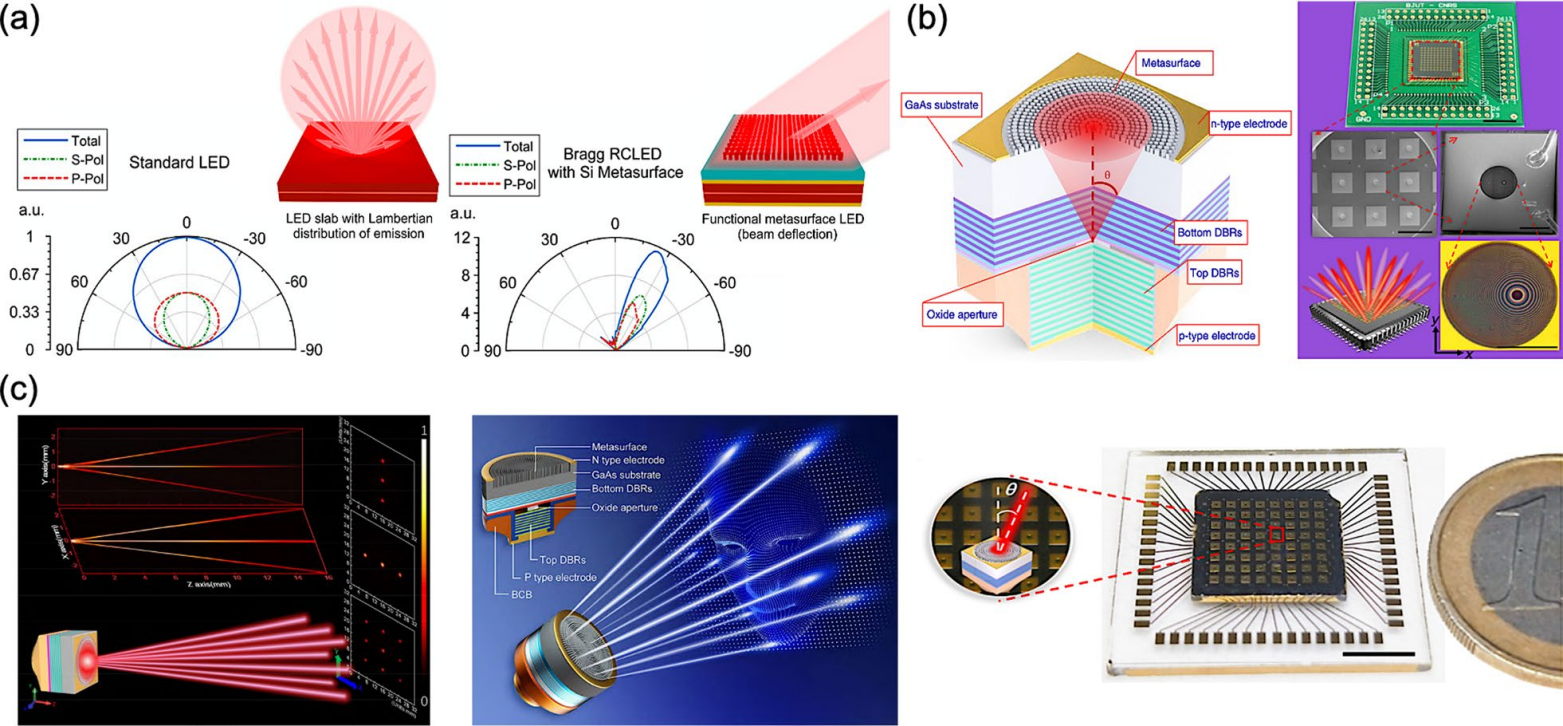
Structured light projection with metasurface-integrated devices **a** Wide angle Lambertian distribution of a plain LED slab (left), Controlled LED emission with functional dielectric metasurfaces (right) [126], **b** Schematic of a standard VCSEL structure integrated with a metasurface at the back side of the substrate (left), Metasurface-integrated VCSELs for programmable directional lasing emissions (right) [142], and **c** Multichannel beams array generations (left), Ultracompact generating structured light on an on-chip integration of metasurfaces and VCSELs (middle), Beam steering chip consisting of 8 × 8 laser elements with different deflection angles (right) [143]

In 2020, E. Khaidarov et al*.* experimentally proposed two integrated light-emitting apparatuses, combining metasurfaces with light-emitting diodes (LEDs) with specific light manipulation capabilities, as shown in Fig. 11a [126]. In the structure of integrated light-emitting apparatus, a hybrid metallic-Bragg cavity is included inside the gallium phosphide (GaP) LED, increasing the overall spatial coherence of light emission through an angular collimation. After that, relevant silicon metasurfaces are fabricated on the top of GaP LEDs, enabling two different functionalities: a directional apparatus for deflected light emission and an apparatus for vortex light emission with an orbital angular momentum. The proposed concept reveals the integration of practical metasurfaces for an unprecedented control of incoherent light. Thus, the experimentally presented solution paves a promising solution toward a multifunctional, efficient, highly integrated meta-device.

For integrating diverse metasurfaces with coherent light sources, as early in 1988, the first room-temperature lasing action in a VCSEL had been demonstrated [127]. Due to the low-power consumption, circular beam profile, mass production in wafer level, and a large-scale two-dimensional array can be fabricated, VCSELs has drawn much attention from then on. Thereby, over the last 30 years, a soaring development utilizing this auspicious coherent light emitter has been done in various applications, such as optical communication, manufacturing, and sensing [128–132]. Moreover, to further improve the beam quality and achieve a brighter light emission, several strategies have been attempted, such as reducing the oxide aperture inside the structure, adding a surface relief structure, incorporat with anti-resonant waveguide structure, etc. Besides, replacing the top reflector with resonant structures, namely photonic crystals, has been exploited as well, and thus forming a photonic crystal surface-emitting laser (PCSEL) [133–137]. For VCSELs, the inherent divergence angle of fundamental single mode usually exceeds 10°; therefore, a further collimating is indeed.

As the aforementioned depth perception system with structured light patterns, the on-demand miniaturization and the integration in relevant optical technologies become more and more prevalent, especially for the portable consumer electronics. Therefore, pioneering effects of the monolithic integration to accomplish desired beam profiles with superior beam quality had been demonstrated [138–141]. However, these DOE-based VCSELs can only operate at low deflection angles and the requirement of multiple phase levels for a complex modulation will increase the complexity of fabrication. Mercifully, the aforementioned emerging metasurfaces can outperform these obstacles with great flexibility in a subwavelength spatial resolution. Thereupon, compared to the conventional optical components, metasurfaces exhibit mass production in wafer level and the complementary MOS fabrication compatibility, making these flat-profile artificial structures prospective candidates for a monolithic integration of ultracompact meta-devices.

In 2020, Y. Y. Xie et al*.* demonstrated a monolithic integration of dielectric metasurfaces with VCSELs, presenting a series of remarkable and versatile modulation, including a collimated laser light emission with various deflection angles, a Bessel laser beam emission, and a vortex laser beam emission with an orbital angular momentum, as shown in Fig. 11b [142]. Thus, for such wafer-level integration, these implementations can simplify the entire assembling process, preserving the device performance as well. Furthermore, to achieve an arbitrary wavefront scanning, the combination of distinct beam-shaping metasurfaces and a two-dimensional VCSEL array configuration can be a prospective solution for the programmable laser beam steering purpose with generation of multi-channel light sources. Consequently, using the state-of-the-art packaging techniques, a multi-channel array of 10 × 10 VCSELs with relevant metasurfaces of different deflection angles is mounted onto a printed circuit board, presenting an electrical tunability for dynamic wavefront shaping with a high operation speed.

Soon in 2021, Q. H. Wang et al*.* implemented a structured light generation through the monolithic integration of metasurface with VCSELs, paving new perspectives of the ultracompact wafer-level designs for structured light projection, as shown in Fig. 11c [143]. Thus, the ultracompact structured laser chips are experimentally demonstrated, realizing versatile functionalities, such as a multi-channel array, an on-chip large-angle beam steering up to 60°, and a remarkably holographic beam shaping with a wide FOV around 124°. Three on-chip beam structuring devices are demonstrated, splitting the emitting laser beams from bottom VCSELs into 1 × 3, 3 × 1, and 3 × 3 output beams arrays, respectively. Besides, a multi-channel chip array consists of 8 × 8 VCSELs with different deflection angles in the range from 0° to 60° is mounted onto a printed circuit board, achieving an arbitrary wavefront scanning. Finally, a wafer-level holographic beam shaping is presented for the constructuin of of complex beam patterns, as a hybridized meta-device with aforementioned functionalities, promoting the development of ultracompact light structuring systems.

## Light-Emitting Metasurface

Miscellaneous pioneering effects in the aforementioned monolithic integration of metasurfaces with light-emitting sources, such as VCSELs, can consequently realize ultracompact meta-devices with versatile functionalities. Besides, the integration technology is compatible with the rapidly emerging of reconfigurable and tunable metasurfaces as well, thus hybridizing the dynamic performance for arbitrary wavefront shaping toward an ultracompact meta-device can be a prospective outcome. However, the integration herein is still a combination of relevant light-emitting sources with passive, or active in the future, metasurfaces. Despite the fact that the combination of light-matter interaction originated from artificial structures and light-emitting materials is still remained, novel schemes and regimes for merging these two aspects into a revolutionary lighting concept are possible. Even for the field of nonlinear optics, relevant metasurfaces can be adopted and integrated as well [45, 46].

**Fig. 12:**
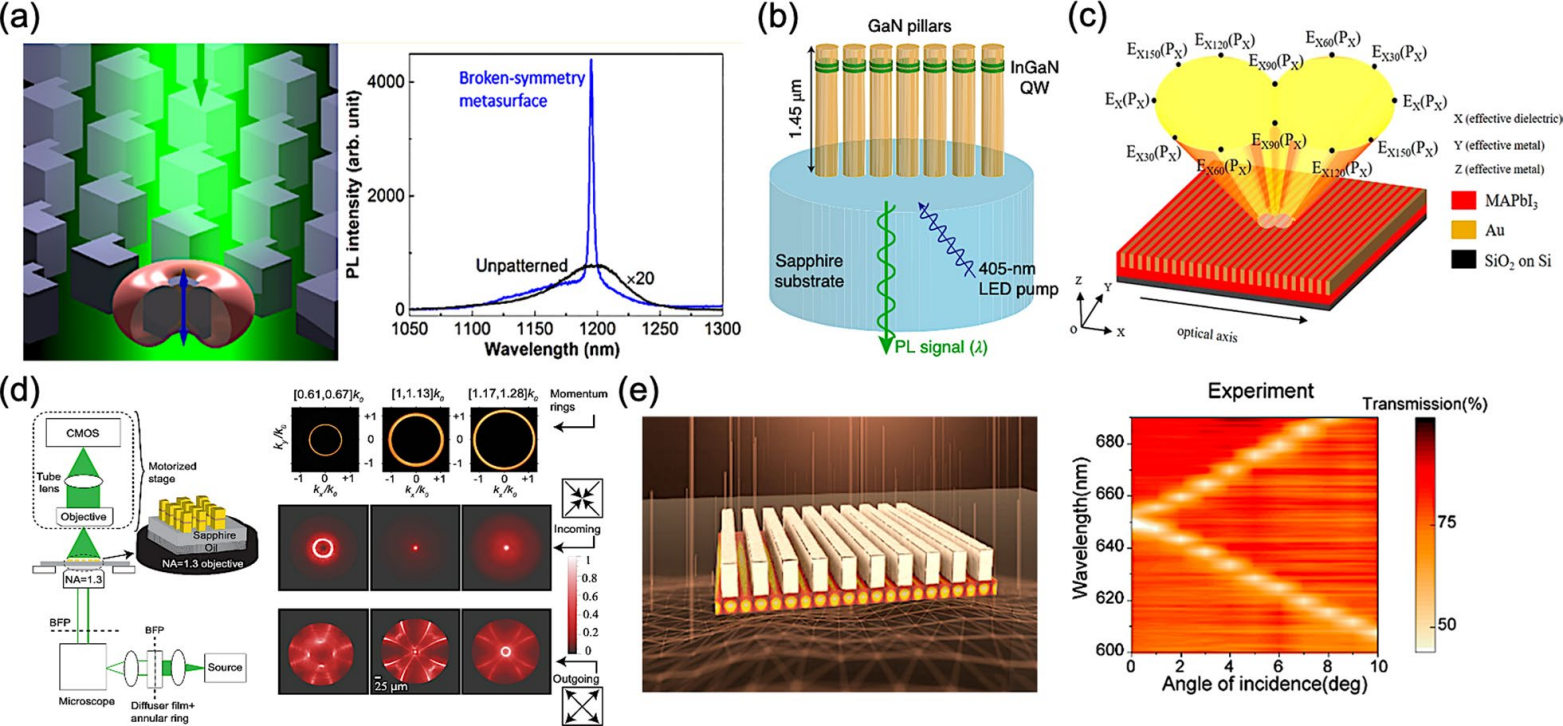
Light-emitting metasurfaces **a** Modal structure of symmetric and symmetry broken semiconductor metasurfaces [47], **b** Unidirectional luminescence from the InGaN/GaN quantum-well metasurface [48], **c** Active perovskite hyperbolic metasurface with deep-subwavelength alternating layers [49], **d** Light-emitting metalenses and meta-axicons for focusing and beaming of spontaneous emission [50], and **e** Enhanced multiphoton processes in perovskite metasurfaces [51]

In 2018, Y. Y. Xie et al*.* proposed a multifunctional and smart lighting device, pairing an all-dielectric metasurface with embedded quantum emitters, as shown in Fig. 12a [47]. Semiconductor heterostructures containing epitaxial quantum dots are fabricated for a Mie-resonant metasurface. Thus, by manipulating the symmetry of resonant modes, namely the overlapping in relevant spectra and other structural parameters, the corresponding light emission can be regulated. As a consequence, the corresponding light emission intensity exhibits a twofold enhancement and a reduction of far-field divergence can be observed as well.

In 2020, P. P. Iyer et al*.* presented the unidirectional PL in the visible region from indium gallium nitride/gallium nitride (InGaN/GaN) quantum-well metasurfaces, overcoming the difficulties due to the lack of phase-locking incident wave of an incoherent light source, as shown in Fig. 12b [48]. Thereby, the metasurface-based quantum-well structures can generate narrow, unidirectional lobes of transmission and emission at arbitrary engineered angles. Furthermore, sevenfold and 100-fold photoluminescence (PL) enhancements of total and air-coupled external quantum efficiency (EQE) are also observed, respectively.

Apart from III-V semiconductor materials, the rapidly emerging perovskite materials are also exploited for their recent progressive development in light-emitting applications and the flexibility of solution-processability with a high degree of freedom in composition tunability [144–146]. Thus, in 2020, Z. Li et al*.* presented an active perovskite hyperbolic metasurface, manipulating electromagnetic waves within the subwavelength scale, as shown in Fig. 12c [49]. Typically, a hyperbolic metasurface is composed of a series of metal-dielectric composites, resulting in an unavoidable optical loss in metals. To compensate this optical loss from metals, perovskite as a gain material is incorporated into the configuration. Therefore, the corresponding metal and dielectric constituents for presented hyperbolic metasurface are gold and perovskite, respectively. As a result, an extreme anisotropy can be observed at around 750 with high degrees of linear polarization, which are greater than 0.8 in both the emission and absorption.

In 2021, Y. Mohtashami et al*.* made a step forward, owing to the adoption of relevant metasurfaces, the incoherent nature of spontaneous emission can be tailored, as shown in Fig. 12d [50]. Two metasurfaces are fabricated for meta-axicons and metalenses, and thus realizing collimated and focused emitting light beams, respectively. Based on these efforts, light sources with arbitrary functionalities can be implemented within the same compact space.

For the multiphoton absorption and luminescence, in 2021, Y. Fan et al*.* demonstrated the enhanced multiphoton processes in perovskite metasurfaces, as shown in Fig. 12e [51]. The proposed perovskite metasurfaces reveal a great efficiency in the nonlinear multiphoton processes, which is comparable to that in linear counterpart. Due to the structured surfaces, the two-photon stimulated emission from the fabricated perovskite metasurface exhibits selection rules of two-photon absorption as well.

Moreover, for the recent progress in quantum sources and quantum information science, metasurface-integrated methods are prosperously developed and demonstrated [147–150]. For the metasurface-integrated quantum sources, in 2020, L. Li et al*.* integrated a metalens array with a nonlinear crystal [147]. Thus, a 100-path spontaneous parametric down-conversionphoton-pair source in a 10 × 10 array can be demonstrated for high-dimensional entanglementand multiphoton-state generation with different phases encoded by metalenses. In 2020, Y. Bao et al*.* accurately integrated a bifocal metalens with quantum dot (QD), depressing the randomicity of the photon streams emitted from the QD [149]. Hence, with the adoption of metalens, the emitted photon streams exhibit the simultaneously on-demand manipulation of the polarization, propagation, and collimation.

## Conclusions

Remarkable progress of reconfigurable and tunable metasurfaces aforementioned are outlined with difference in active component, operation spectrum, modulation speed, advantages and limitation. Different types of modulation mechanisms and properties mentioned in this review paper are compared in Table 1. Based on the comparison, people can select suitable metasurface for their purpose shortly.

**Table 1:** Comparison of multiple mechanisms for modulation

Modulation mechanism	Active component	Operation spectrum	Modulation speed	Advantages	Limitations	Refs
Mechanical tunability	Au/**PDMS** Cu w/o substrate Al/**PDMS** Au/**PDMS** Al/**PDMS**	Vis Microwave Vis Vis MIR	s to ms scale	Simple, low cost, extensive tuning range	Modulation speed, stability, reliability	[52–56]
Thermal tunability	**VO**_**2**_/Au/sapphire Au/SiO_2_/**VO**_**2**_/Si **VO**_**2**_/sapphire **VO**_**2**_/Si *h*BN/**VO**_**2**_/quartz	NIR to MIR mm *μ*m MIR MIR	ms scale	Reversible, low transition temperature	Low heating speed, energy loss	[65–69]
	**GST**/Al/Si Al/**GST**/Al/Si Au/**GST**/CaF_2_ ZnS/SiO_2_-**GST**-ZnS/SiO_2_/quartz **GST**/CaF_2_	NIR to MIR NIR to MIR NIR to MIR Vis NIR to MIR	ms to fs scale	Efficient, stable, nonvolatile	Cooling rate limited, energy loss	[72–76]
Optical tunability	**nGaAs**/AlGaAs/epoxy/sapphire Al/**Si**/SiO_2_ **Si**/sapphire All **Si**	THz	fs to ps scale	Ultrafast speed, nonvolatile	Power consumption, modulation depth	[78–81]
Electrical tunability	Au/**ITO**/SiO_2_/Au/quartz **ITO**/Au/SiN_x_/Si/Au Au/Al_2_O_3_/**ITO**/Au/quartz Au/Al_2_O_3_/**ITO**/Au Si/Al_2_O_3_/**ITO**/Au/Si Au/HAOL/**ITO**/Al_2_O_3_/Au/PCB	Vis to MIR NIR to MIR NIR THz THz NIR	μs scale	Fast speed, good stability	Power consumption, modulation depth	[32, 35–37, 98, 99]
	**ITO**/Si/fused silica Au/Al_2_O_3_/**ITO**/Si	NIR NIR	μs scale	Fast speed, good stability	Power consumption, modulation depth	[38, 39]

The significance of bold indicates the corresponding tunable components in metasurfaces for different types of modulation mechanisms

Nevertheless, pioneering effects in the monolithic integration combine metasurfaces with light-emitting sources, the aforementioned integration approaches are still a combination of relevant light-emitting sources with passive, or active in the future, metasurfaces. However, the combination of light-matter interaction originated from artificial structures and light-emitting materials is still remained. Novel schemes and regimes for merging these two aspects into a revolutionary lighting concept are possible. Several works are therefore demonstrated, realizing a direct light-emitting meta-device with arbitrary functionalities.

These demonstrated paradigms promise pavements of active metasurfaces with tunability and reconfiguration, monolithic integration with modern semiconductor technology, and even the revolutionary light-emitting metasurfaces, unprecedented merits with new functionality over conventional optical components can be obtained with arbitrary functionalities. These state-of-the-art metasurfaces open a path to a new type of platform for future ultracompact nanophotonics with metasurfaces and lay promising groundwork of consumer electronics utilizing metaphotonics in the future decades.

## Data Availability

Not applicable.

## Abbreviations

IoT: Internet of things
AR: Augmented reality
VR: Virtual reality
MOSFET: Metal-oxide-semiconductor field-effect transistor
MOS: Metal-oxide-semiconductor
Au: Gold
PDMS: Polydimethylsiloxane
SPP: Surface plasmon polariton
MEMS: Micro-electro-mechanical-system
PCM: Phase change material
VO_2_: Vanadium dioxide
GST: Germanium-antimony-tellurium (Ge:Sb:Te)
GHz: Gigahertz
Al_2_O_3_: Alumina
FPGA: Field-programmable gate array
*h*BN: Hexagonal boron nitride
THz: Terahertz
fs: Femtosecond
ps: Picosecond
GaAs/AlGaAs: Gallium arsenide/aluminium gallium arsenide
BJT: Bipolar junction transistor
FET: Field-effect transistor
MIM: Metal-insulator-metal
TCO: Transparent conducting oxide
ENZ: Epsilon-near-zero
ITO: Indium-tin oxide
ZnO: Zinc oxide
CdO: Cadmium oxide
2D: Two-dimensional
DOE: Diffractive optical element
VCSEL: Vertical-cavity surface-emitting laser
FOV: Field of view
OPL: Optical path length
LED: Light-emitting diode
GaP: Gallium phosphide
PCSEL: Photonic crystal surface-emitting laser
PL: Photoluminescence
InGaN/GaN: Indium gallium nitride/gallium nitride
EQE: External quantum efficiency
OAM: Orbital angular momentum
QD: Quantum dot
